# Supplementary figures and images for: Bicine promotes rapid formation of β-sheet-rich amyloid-β fibrils
Source: PLoS One. 2020 Oct 13;15(10):e0240608. doi: 10.1371/journal.pone.0240608 (PMC7553346; doi:10.1371/journal.pone.0240608)

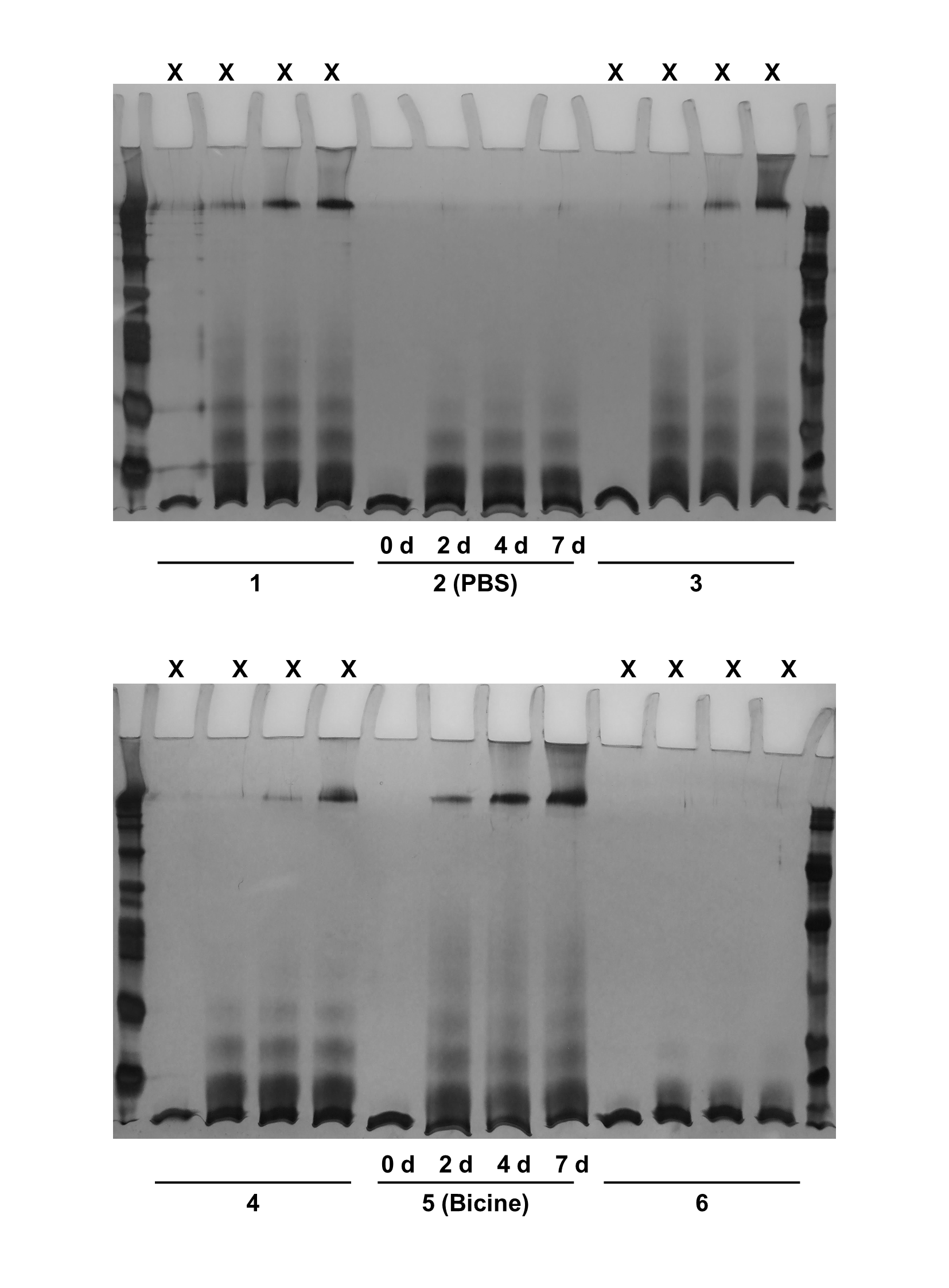

Supplement: S1 Fig — Aβ(1–40) (50 μM) peptides were dissolved and incubated in 6 different buffers, including bicine (20 mM), PBS (1X) and 4 unreported compounds (1, 3, 4, and 6), at 3 time points: 2, 4 and 7 days. The 0 day Aβ(1–40) samples were dissolved in the same buffers and immediately stored at −70°C before use. Aβ aggregates were visualized by silver staining after SDS-PAGE with PICUP. These experiments were performed in triplicate independently. SDS-PAGE: sodium dodecyl sulfate-polyacrylamide gel electrophoresis, PICUP: photo-induced cross-linking of unmodified proteins, d: day. (TIF) [file pone.0240608.s001.TIF]

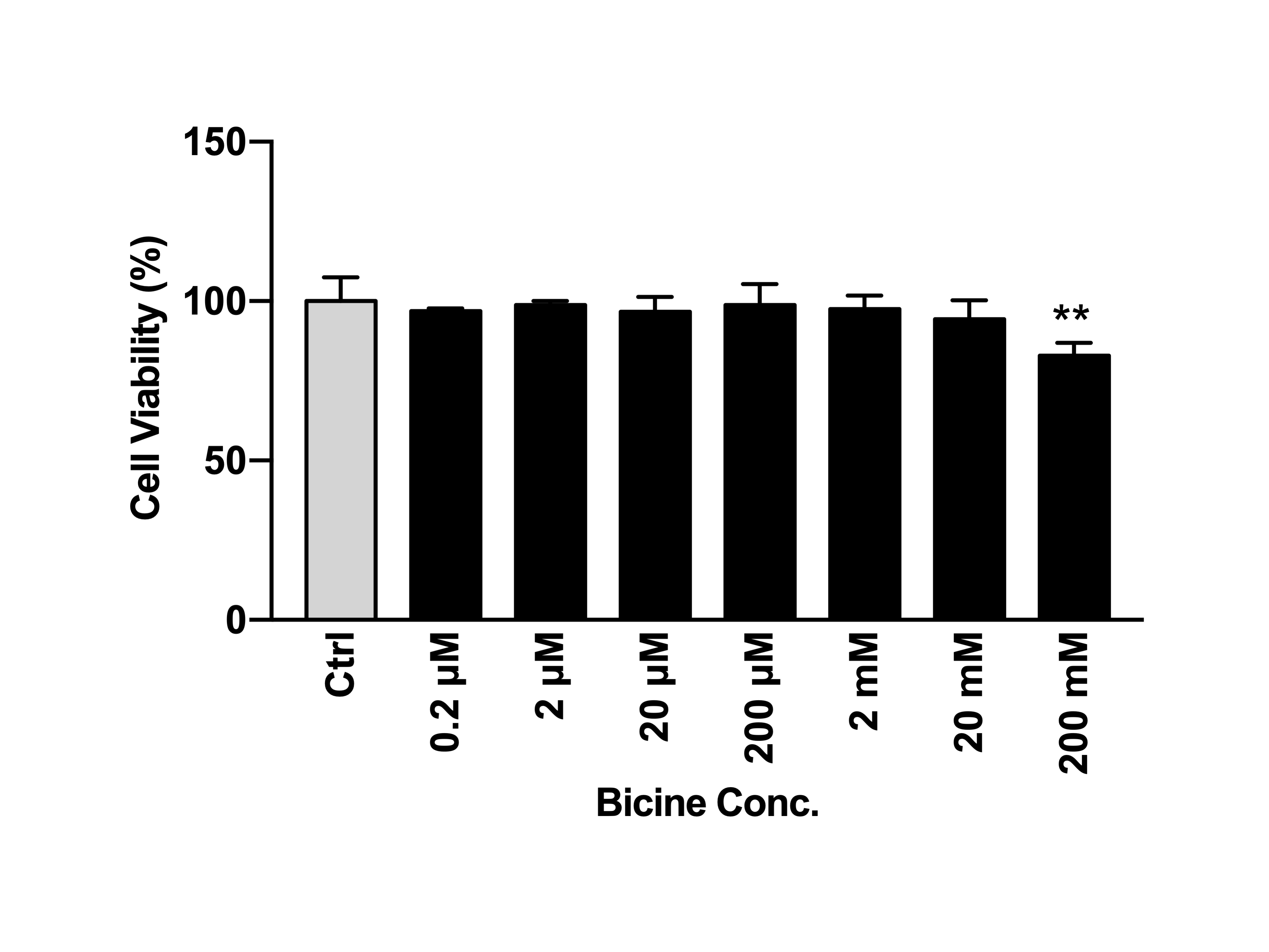

Supplement: S2 Fig — Assessing bicine-induced cytotoxicity via MTT cell viability assay. Increasing concentrations of bicine was treated to HT-22 cells. Non-treated cells were used as a control. These experiments were performed in triplicate independently. Error bars indicate the standard deviation. Statistical analysis was performed using one-way ANOVA followed by Tukey’s multiple comparison test. **P ≤ 0.01. Conc.: concentration, d: day, Ctrl: control, d: day. (TIF) [file pone.0240608.s002.TIF]
