# Supplementary material for: Bicine promotes rapid formation of β-sheet-rich amyloid-β fibrils
Source: PLoS One. 2020 Oct 13;15(10):e0240608. doi: 10.1371/journal.pone.0240608 (PMC7553346; doi:10.1371/journal.pone.0240608)
Supplement: Attachment — Submitted filename: Response to Reviewers.docx [file pone.0240608.s003.docx]

Response to Editors and Reviewers

Dear Editor,

Thank you for the timely review of our manuscript (PONE-D-20-20947) entitled, “**Bicine promotes rapid formation of β-sheet-rich amyloid-β fibrils”** for potential publication in *PLOS ONE*. We appreciate this opportunity to revise our manuscript and helpful comments from the reviewers.

Original uncropped and unadjusted images of gels are now provided in Supporting Information. Also, figure 3 was adjusted accordingly to gel reporting requirements of PLOS ONE. As our experiment did not use any human or animal subjects and/or tissue, we did not provide an ethics statement.

Here, we provide a detailed point-by-point response to the reviewer’s comments below. The font styles are following:

**Reviewer’s comments are in bold black and numbered**.

Authors’ responses are in black.

Sentences from original manuscript are in gray highlighted.

Sentences from revised manuscript are in yellow highlighted.

We thank you for your consideration and hope that you find the revised paper suitable for publication in *PLOS ONE*.

Sincerely,

YoungSoo Kim, PhD

Associate Professor

Department of Pharmacy

Yonsei University

85 Songdogwahak-ro, Yeonsu-gu, Veritas Hall D411

Incheon 21983, South Korea

Phone: +82-32-749-4523

E-mail: [y.kim@yonsei.ac.kr](mailto:y.kim@yonsei.ac.kr)

**Response to Reviewer’s Comments:**

**Minor Comments**

1. **Ln. 137, 153 and elsewhere: “…serial concentrations of bicine (0.07813 to 20 mM)…” — the precision of concentrations must be expressed uniformly, to a certain number of significant digits.**

- Thank you for your comments. We have accordingly changed the expression of significant figures in a uniform manner.

Ln. 86: “For the concentration-dependent assay, Aβ_40_ (50 μM) was incubated in serial concentrations of bicine (78.13, 156.25, 312.5, 625, 1250, 2500, 5000, 10000, 20000 μM).”

Ln. 86: “For the concentration-dependent assay, Aβ_40_ (50 μM) was incubated in serial concentrations of bicine (78.130, 156.25, 312.50, 625.00, 1250.0, 2500.0, 5000.0, 10000, 20000 μM).”

Ln. 135: “To analyze the propensity of amyloid fibril formation in increasing buffer concentrations, we incubated monomeric Aβ_40_ peptides (50 μM) in serial concentrations of bicine (0.07813 to 20 mM) at 37°C for 2 days.”

Ln. 136: “To analyze the propensity of amyloid fibril formation in increasing buffer concentrations, we incubated monomeric Aβ_40_ peptides (50 μM) in serial concentrations of bicine (78.130 μM to 20.000 mM) at 37°C for 2 days.”

Ln. 152: “Aβ(1-40) (50 μM) peptides were dissolved and incubated in serial concentrations of bicine (78.13, 156.25, 312.5, 625, 1250, 2500, 5000, 10000, 20000 μM, presenting ln concentration) for 2 days at 37°C.”

Ln. 153: “Aβ(1-40) (50 μM) peptides were dissolved and incubated in serial concentrations of bicine (78.130, 156.25, 312.50, 625.00, 1250.0, 2500.0, 5000.0, 10000, 20000 μM, presenting ln concentration) for 2 days at 37°C.”

2. **Ln. 155 and elsewhere — the PBS concentration is not disclosed in the manuscript.**

- Thank you for your comment. We have added the concentration of PBS (1X) to the materials and methods and figure legend sections in this manuscript.

Ln. 68: “PBS (1X) consists of phosphate buffer concentration of 0.01M and a sodium chloride concentration of 0.154M.” (Added)

Ln. 154: “Aβ(1-40) (50 μM) peptides were dissolved and incubated in bicine (20 mM) or PBS at 3 time points: 2, 4 and 7 days.”

Ln. 155: “Aβ(1-40) (50 μM) peptides were dissolved and incubated in bicine (20 mM) or PBS (1X) at 3 time points: 2, 4 and 7 days.”

Ln. 175: “Aβ(1-40) (25 μM) peptides were dissolved and incubated in bicine (20 mM) for 2 days or PBS for 0 and 2 days.”

Ln. 176: “Aβ(1-40) (25 μM) peptides were dissolved and incubated in bicine (20 mM) for 2 days or PBS (1X) for 0 and 2 days.”

Ln. 203: “Aβ(1-40) (50 μM) peptides were dissolved and incubated in bicine (20 mM) or PBS at 3 time points: 2, 4 and 7 days.”

Ln. 204: “Aβ(1-40) (50 μM) peptides were dissolved and incubated in bicine (20 mM) or PBS (1X) at 3 time points: 2, 4 and 7 days.”

3. **Fig. 2. The figure’s title must relate to both buffers used, not to bicine only.**

- Thank you. We have inserted “PBS” into the title of Fig. 2.

Ln. 174: “Fig 2. Analysis of Aβ(1-40) peptides in bicine via (a) structural analysis by CD spectra and (b) TEM imaging.”

Ln. 175: “Fig 2. Analysis of Aβ(1-40) peptides in bicine and PBS via (a) structural analysis by CD spectra and (b) TEM imaging.”
